# Effects of Pulsed Electromagnetic Field Treatment on Skeletal Muscle Tissue Recovery in a Rat Model of Collagenase-Induced Tendinopathy: Results from a Proteome Analysis

**DOI:** 10.3390/ijms25168852

**Published:** 2024-08-14

**Authors:** Enrica Torretta, Manuela Moriggi, Daniele Capitanio, Carlotta Perucca Orfei, Vincenzo Raffo, Stefania Setti, Ruggero Cadossi, Laura de Girolamo, Cecilia Gelfi

**Affiliations:** 1 Laboratory of Proteomics and Lipidomics, IRCCS Orthopedic Institute Galeazzi, 20161 Milan, Italy; cecilia.gelfi@unimi.it; 2 Department of Biomedical Sciences for Health, University of Milan, 20090 Segrate, Italy; manuela.moriggi@unimi.it (M.M.); daniele.capitanio@unimi.it (D.C.); 3 Orthopaedic Biotechnology Laboratory, IRCCS Orthopedic Institute Galeazzi, 20161 Milan, Italyvincenzo.raffo@grupposandonato.it (V.R.); laura.degirolamo@grupposandonato.it (L.d.G.); 4 IGEA Clinical Biophysics, 41012 Carpi, Italy

**Keywords:** skeletal muscle, PEMF, tendinopathy, proteomics, recovery

## Abstract

Tendon disorders often result in decreased muscle function and atrophy. Pulsed Electromagnetic Fields (PEMFs) have shown potential in improving tendon fiber structure and muscle recovery. However, the molecular effects of PEMF therapy on skeletal muscle, beyond conventional metrics like MRI or markers of muscle decline, remain largely unexplored. This study investigates the metabolic and structural changes in PEMF-treated muscle tissue using proteomics in a rat model of Achilles tendinopathy induced by collagenase. Sprague Dawley rats were unilaterally induced for tendinopathy with type I collagenase injection and exposed to PEMFs for 8 h/day. *Gastrocnemius* extracts from untreated or PEMF-treated rats were analyzed with LC-MS/MS, and proteomics differential analysis was conducted through label-free quantitation. PEMF-treated animals exhibited decreased glycolysis and increased LDHB expression, enhancing NAD signaling and ATP production, which boosted respiratory chain activity and fatty acid beta-oxidation. Antioxidant protein levels increased, controlling ROS production. PEMF therapy restored PGC1alpha and YAP levels, decreased by tendinopathy. Additionally, myosins regulating slow-twitch fibers and proteins involved in fiber alignment and force transmission increased, supporting muscle recovery and contractile function. Our findings show that PEMF treatment modulates NAD signaling and oxidative phosphorylation, aiding muscle recovery through the upregulation of YAP and PGC1alpha and increasing slow myosin isoforms, thus speeding up physiological recovery.

## 1. Introduction

Tendon disorders are very common in sports as well as in the general population, often leading to severe complications such as contractures, adhesions, muscle wasting, and disability. These injuries negatively impact adjacent tissues, including joints, skeletal muscles, and bones. Notably, muscular changes following tendon tears can be attributed to the interplay between the tendon and muscle at the musculotendinous junction [1,2,3], resulting in atrophy and loss of elasticity after tendon rupture [4,5]. Clinical treatments aim not only to restore tendon functionality but also to rehabilitate the entire muscle–tendon–bone unit. Among conservative treatments, Pulsed Electromagnetic Fields (PEMFs), which induce an electric current in tissues with rapid magnetic pulse field changes [6], are considered a promising therapy for tendon disorders. The use of PEMFs was approved by the FDA in 1979 as a non-invasive medical treatment for non-union fractures. Since then, electromagnetic fields have been tested for various musculoskeletal conditions, including articular cartilage defects [7] and osteoarthritis (OA) [8], with satisfactory results. It has been demonstrated that PEMFs inhibit the release of prostaglandin E2 (PGE2) and pro-inflammatory cytokines interleukin-6 (IL-6) and interleukin-8 (IL-8) in human synovial fibroblasts. These effects are mediated by the PEMF-induced upregulation of adenosine A2A receptors (A2ARs) [9]. Electromagnetic stimulation promotes the migration of fibroblast-like synoviocytes to accelerate cartilage repair in vitro [10] and enhances cartilage growth in in vivo models [11,12]. PEMFs preserve the morphology of articular cartilage and slow the progression of OA lesions on the articular surface of the knee in aged osteoarthritic guinea pigs, exerting a chondroprotective effect [13]. Electromagnetic fields also decrease IL-6 and TNF-α expression in cartilage and have positive effects on pain, cartilage degeneration, and synovitis in mice [14]. Regarding bone, most studies have addressed the use of PEMFs for treating osteoporosis [15] and preventing the risk of fractures [16,17] by improving bone density and mass, stimulating osteoblastogenesis, and modulating calcium storage and mineral metabolism. Other studies have shown that PEMFs can improve osteoblast differentiation through nitric oxide and Ca^2+^/NO/cGMP/PKG signaling [18,19]. In human mesenchymal stem cells derived from bone marrow stroma, Petecchia et al. demonstrated that the PEMF effect is primarily associated with an early enhancement in intracellular calcium concentration, proposed as a reliable hallmark of the osteogenic developmental stage [20].

In a cell culture of healthy human tenocytes, it has been demonstrated that PEMFs positively influence cell proliferation, tendon-specific marker expression, and the release of anti-inflammatory cytokines and angiogenic factors [21]. Varani et al. [22] introduced the role of PEMFs in the activity of A2As and A3ARs expressed in various cells and tissues, demonstrating that their activation promotes a reduction in pro-inflammatory cytokines. The role of A2ARs was recently confirmed in human tenocytes exposed to PEMFs for 48 h, where electromagnetic fields induced an anabolic and reparative response [23]. The application of PEMFs promoted tendon recovery in young rats, inducing fiber alignment and a reduction in inflammatory cytokines [24]. On muscle, the effect of PEMFs was mainly assessed on patient recovery after surgery [25], to counteract muscle soreness [26], and recently, in improving muscle performance in semi-professional cycling athletes [27]. A recent study demonstrated an improvement in the physical performance and lean body mass of old healthy subjects [28]. Collectively, these studies determined the efficacy of PEMFs by measuring muscle performance or systemic metabolism recovery by MRI or following known markers of muscle decline in serum [25]. More detailed information associated with PEMF effects in muscle has been introduced by a study in mice exposed to PEMFs (10 min/week) for a few months and in a recent study on skeletal muscle cell regeneration after injury [29]. The results indicated an enhancement in oxidative muscle metabolism and fatty acid oxidation and an improvement in insulin sensitivity [30] as well as an increase in antioxidant enzymes [29]. Overall, the effects of PEMFs on muscle tissue are still debated, and their outcomes at the molecular level remain under-investigated.

In muscles, metabolism plays a pivotal role in providing substrates for energy production and contraction. Metabolic changes impact muscle function and can prolong or reduce the time required for muscle recovery. Proteomics allows for the identification of differential changes in the majority of proteins expressed in muscle tissue, providing a snapshot of structural, contractile, and extracellular matrix protein composition, as well as molecules involved in cell signaling and in the metabolic support of muscle function [31].

The present study is based on liquid chromatography with tandem mass spectrometry (LC-MS/MS) differential analysis of the *gastrocnemius* muscle after collagenase-induced tendinopathy in young rats treated or untreated with PEMFs. The aim of the present study is to elucidate the impact at the molecular level of tendinopathy on muscle tissue and the beneficial effect of PEMFs on muscle recovery.

In light of the 3Rs principle (replacement, reduction, and refinement), muscle tissue was recovered from the limbs of rats involved in a study aimed at investigating the effects of PEMFs on tendon tissue after collagenase-induced tendinopathy [24]. The total-body exposure of the animals to PEMFs made it possible to analyze the effect of PEMFs on multiple tissues, including muscle, without sacrificing additional animals.

## 2. Results

**Figure 1:**
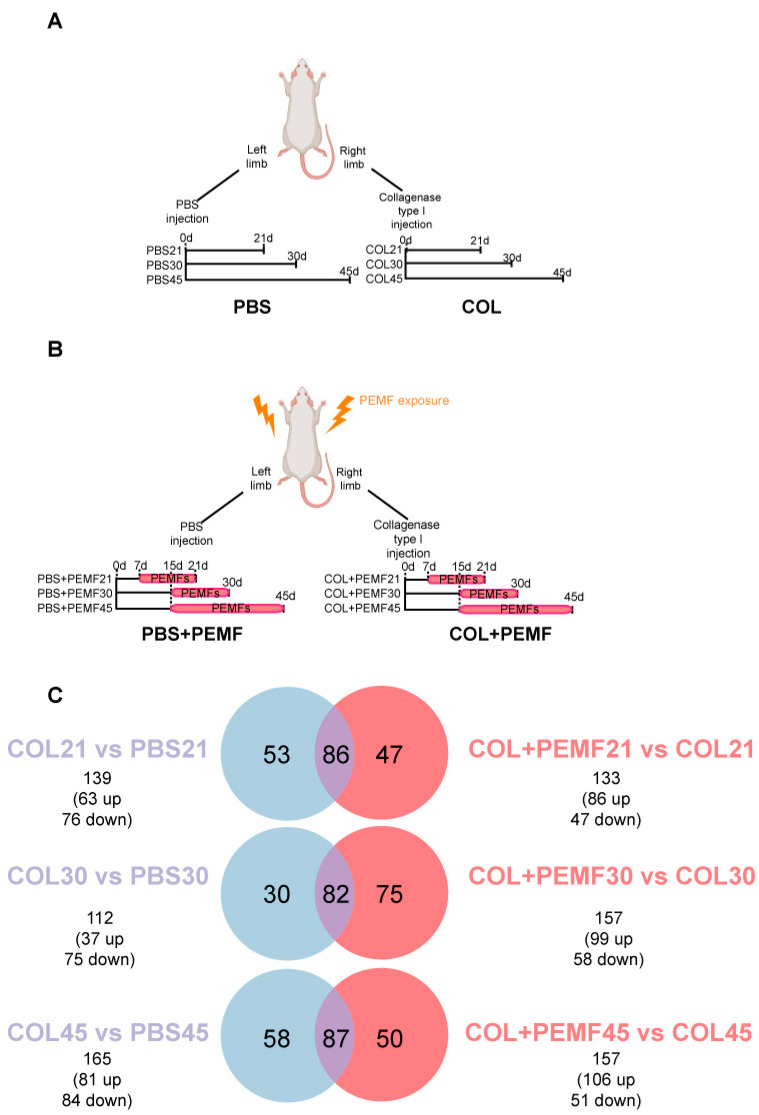
Experimental design and label-free LC–ESI–MS/MS results. (**A**) Untreated rats. Type I collagenase was injected into the right limbs (COL), while PBS was injected into their left counterparts (PBS). Animals were sacrificed on day 21, 30, and 45 after the injection. Based on this, right muscles (COL) were named COL21, COL30, and COL45, while left muscles (PBS) were named PBS21, PBS30, and PBS45. (**B**) PEMF-treated rats. Animals were treated with PEMFs (1.5 mT SD 0.2; 75 Hz) for eight hours/day. Right muscles (COL + PEMF) were named COL + PEMF21, COL + PEMF30, and COL + PEMF45. (**C**) Venn diagram. Comparisons of shared and distinct significantly changed proteins in COL vs. PBS (dusty blue circles) and in COL + PEMF and COL (pink circles). Data resulted from label-free quantitation after LC–ESI–MS/MS analysis (ANOVA followed by Tukey’s multiple comparison test, *p*-value < 0.05). The graphical illustration was generated using BioRender (version 4).

To investigate the impact of Achilles tendinopathy on the *gastrocnemius* muscle and the effects of PEMF treatment on muscle function and recovery, protein extracts from the *gastrocnemius* were analyzed by liquid chromatography coupled to electrospray tandem mass spectrometry (LC–ESI–MS/MS) and quantified by a label-free approach. Comparing right limbs (in which type I collagenase was injected) vs. their left counterparts (in which PBS was injected) (COL vs. PBS), 139 proteins were differentially expressed in muscle from injured limbs 21 days after surgery (COL21 vs. PBS21), 112 after 30 days (COL30 vs. PBS30), and 165 after 45 days (COL45 vs. PBS45). Comparing PEMF-treated or untreated injured limbs (COL + PEMF vs. COL), proteome analysis revealed that after 15 days of PEMF treatment, started on day 7 after collagenase injection (COL + PEMF21 vs. COL21), 133 proteins were differentially expressed, whereas 157 proteins were changed after 15 days of PEMF treatment started on day 15 (COL + PEMF30 vs. COL30). The same number of changed proteins was observed when PEMF treatment, started on day 15, was extended to 30 days (COL + PEMF45 vs. COL45) (Table 1 and Figure 1). An ANOVA test followed by Tukey’s multiple comparison post hoc test with *p*-value < 0.05 was adopted for data analysis. Identification data for changed proteins are shown in Supplementary Table S1 for comparisons COL21 vs. PBS21 and COL + PEMF21 vs. COL21, Table S2 for comparisons COL30 vs. PBS30 and COL + PEMF30 vs. COL30, and Table S3 for comparisons COL45 vs. PBS45 and COL + PEMF45 vs. COL45.

### 2.1. Glucose Metabolism and Stress Response

**Figure 2:**
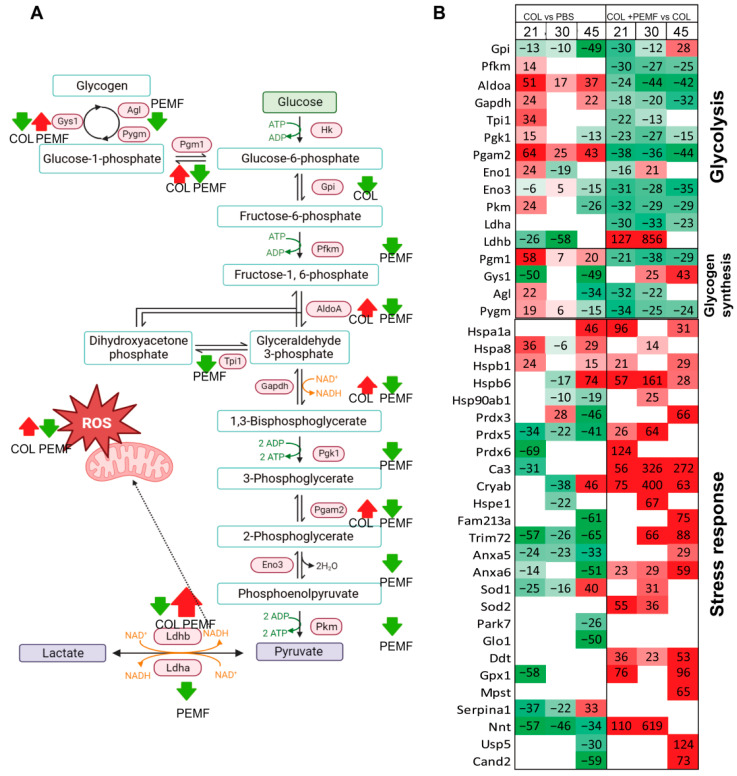
Glucose and stress metabolism. (**A**) Summary of changed metabolic enzymes in glucose metabolism in muscles attached to inflamed tendons, both without (COL) and with PEMF treatment (PEMF). (**B**) Heatmap illustrating the expression profile of significantly increased (in red) or decreased (in green) enzymes (ANOVA and Tukey’s test, *p*-value < 0.05) involved in glucose metabolism and stress response pathways. The graphical illustration was generated using BioRender (version 4).

Figure 2 shows the protein variation in the glycolytic and glycogenesis pathways and in the stress response. An increase in aldolase A (AldoA), glyceraldehyde-3-phosphate dehydrogenase (Gadph), and phosphoglycerate mutase 2 (Pgam2) was observed in injured limbs (COL vs. PBS), together with a decrease in Glucose-6-Phosphate Isomerase (Gpi) and lactate dehydrogenase B (Ldhb) (Figure 2A). Phosphofructokinase (Pfkm), Triosephosphate Isomerase 1 (Tpi1), Phosphoglycerate Kinase 1 (Pgk1), Enolase 1 (Eno1), Pyruvate Kinase (Pkm) increased in the COL21 vs. PBS21 comparison, only (Figure 2B). In contrast, a general decrease in the glycolytic pathway was observed in PEMF-treated vs. untreated injured limbs (COL + PEMF vs. COL). Ldhb was the only glycolytic enzyme that largely increased after PEMF treatment.

In the glycogen biosynthetic pathway, phosphoglucomutase-1 (Pgm1) increased in COL limbs but decreased in COL + PEMF, whereas Glycogen Synthase 1 (Gys1) showed the opposite trend (Figure 2A). Glycogen debranching enzyme (Agl) and Alpha-1,4 glucan phosphorylase (Pygm) initially increased in COL limbs but decreased after 45 days, whereas they were decreased after PEMF treatment (Figure 2B).

To counteract ROS production, a widespread increase in stress response proteins was detected after PEMF treatment (Figure 2B). In the injured limbs of PEMF-untreated rats, a decrease was noticed, except for in Hspa8 and Hspb1 and in Hspa1a, Hspb6, Cryab, Sod1, and Serpin1 after 45 days. LFQ intensity % variations are reported in Supplementary Table S4.

### 2.2. TCA Cycle, Fatty Acid Oxidation, and Oxidative Phosphorylation Pathways

**Figure 3:**
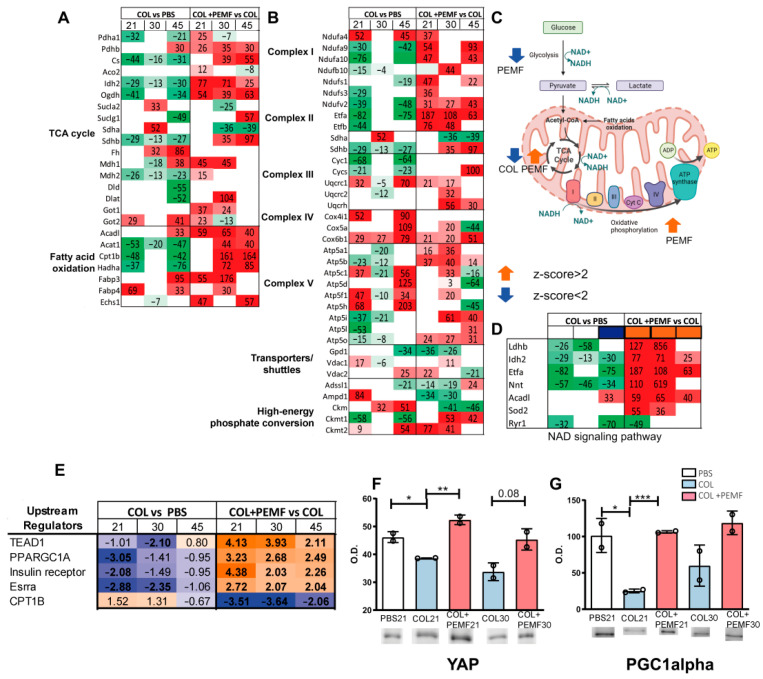
TCA cycle, fatty acid oxidation, and oxidative phosphorylation pathways. (**A**,**B**) Heatmaps illustrating the expression profile of significantly increased (in red) or decreased (in green) enzymes (ANOVA and Tukey’s test, *p*-value < 0.05) involved in TCA cycle, fatty acid oxidation (**A**), and oxidative phosphorylation (**B**) pathways. (**C**) Graphical representation of metabolic canonical pathways: activated (z-score > 2; orange arrows) or inhibited (z-blue < 2; blue arrows) in muscles attached to inflamed tendons, without (COL) and with PEMF treatment (PEMF). (**D**) Heatmap displaying the expression profile of enzymes involved in the NAD signaling pathway. (**E**) Heatmap presenting the most significant upstream regulators. Orange- and blue-colored rectangles indicate predicted regulator activation or inhibition, respectively, via the z-score statistic. (**F**,**G**) Bar graphs depicting the expression of Yes-Associated Protein (YAP) (**F**) and peroxisome proliferator-activated receptor gamma coactivator 1-alpha (PGC1alpha) (**G**) in the *gastrocnemius* muscle from PBS, COL, and COL + PEMF groups (mean ± SD; * = significant difference, ANOVA and Tukey’s test, *n* = 2, * *p*-value < 0.05; ** *p*-value < 0.01; *** *p*-value < 0.001). Full-length images are available in Supplementary Figure S1. The graphical illustration was generated using BioRender (version 4).

In the TCA cycle (Figure 3A), citrate synthase (Cs), isocitrate dehydrogenase (Idh2), 2-oxoglutarate dehydrogenase (Ogdh), succinate dehydrogenase iron–sulfur subunit (Sdhb), and malate dehydrogenase 2 (Mdh2) were decreased in injured vs. control limbs (COL vs. PBS). In contrast, they were increased in PEMF-treated injured limbs (COL + PEMF vs. COL). Conversely, succinyl-CoA ligase subunit beta (Sucla2) and succinate dehydrogenase flavoprotein subunit (Sdha) were increased in COL30 compared to PBS30 but decreased in COL + PEMF30 compared to COL30 and in COL + PEMF45 compared to COL45. Fumarate hydratase (Fh) was increased in COL30 and COL45 compared to PBS30 and PBS45, but no significant changes were detected in the COL + PEMF groups. Malate dehydrogenase 1 (Mdh1), converting NAD+ and malate to NADH and oxaloacetate in the citric acid cycle, increased after 45 days in COL vs. PBS, whereas it was promptly increased in PEMF-treated animals.

In injured untreated rats, the fatty acid pathway (Figure 3A) was characterized by decreased levels of acetyl-CoA acetyltransferase (Acat1), carnitine O-palmitoyltransferase 1 (Cpt1b), and trifunctional enzyme subunit alpha (Hadha), whereas the levels of these enzymes were increased in PEMF-treated rats, becoming significant in the COL + PEMF30 vs. COL30 and COL + PEMF45 vs. COL45 comparisons. Long-chain acyl-CoA dehydrogenase (Acadl) and fatty acid binding protein 3 (Fabp3) increased after 45 days in the COL limb muscle, whereas they increased after 21 days in PEMF-treated rats.

In the oxidative phosphorylation pathway (Figure 3B), several components of the I, II, and III complexes of the mitochondrial respiratory chain were decreased in COL vs. PBS at all time points, except for NADH Dehydrogenase (Ubiquinone) 1 Alpha Subcomplex component 4 (Ndufa4), Sdha, and complex III component cytochrome b-c1 complex subunits 1 (Uqcrc1). Conversely, comparing PEMF-treated vs. untreated rats, an increase in the above-cited components was observed, except for Sdha. In complex V, components of ATP synthase subunit alpha, beta, e, g, and O (Atp5a1, Atp5b, Atp5i, Atp5l, Atp5o) decreased in injured limbs and increased after PEMF treatment. Components of ATP synthase subunit gamma, delta, B1, and D (Atp5c1, Atp5d, Atp5f1, Atp5h) increased at 21 and 45 days, while they decreased after 30 days of PEMF treatment. Adenosine monophosphate deaminase 1 (Ampd1) and creatine kinase, m-type (Ckm), increased in untreated muscles but decreased after PEMF treatment, whereas creatine kinase u-type (Ckmt1), responsible for the transfer of high-energy phosphate from the mitochondria to the cytosol, decreased in untreated rats but increased in treated animals.

### 2.3. Bioinformatic Analysis of Metabolic Pathways

The canonical pathway analysis conducted by IPA software indicated that glycolysis was decreased after PEMF treatment, mostly in COL + PEMF21 vs. COL21 (z-score −3.16) but consistent also in COL + PEMF30 vs. COL30 (z-score −2.53) and COL + PEMF45 vs. COL45 (−2.12). The TCA cycle was decreased in untreated rats sacrificed after 21 days (COL + PEMF21 vs. COL21) (z-score −2) but increased after PEMF treatment (z-score 2). Oxidative phosphorylation was increased in the muscle of injured rats that underwent PEMF treatment, particularly in the COL + PEMF21 vs. COL21 and COL + PEMF30 vs. COL30 comparisons (z-score 3.36) (Figure 3C). This is supported by the evidence of a decrease in the NAD signaling pathway (−2.24) in COL vs. PBS after 21 days, suggesting that restrictions in NAD bioavailability, impairing the production of ATP, decrease the oxidative capacity of the muscle. Conversely, in COL + PEMF vs. COL, enzymes that require NAD as a coenzyme such as LDHB, IDH2, and ETFA were increased, enhancing oxidative metabolism (Figure 3D). A complete list of observed canonical pathways is provided in Supplementary Table S5. Upstream regulator analysis performed by IPA software allows the identification of upstream factors associated with protein expression changes shown in our dataset. TEA Domain Transcription Factor 1 (TEAD1), peroxisome proliferator-activated receptor gamma coactivator 1-alpha (Ppargc1a or PGC1alpha), and insulin receptor and estrogen-related receptor alpha (ESRRA) were predicted to be inhibited in injured limbs but activated after PEMF treatment. In contrast, carnitine palmitoyltransferase 1B (Cpt1b) was predicted to be inhibited in PEMF-treated groups (Figure 3E). To validate the increment in the first two upstream regulators, the levels of YAP (which forms a heterodimer with TEAD1) and of PGC1alpha were assessed by immunoblotting. In inflamed muscle, the levels of YAP were significantly decreased compared to the control group (ANOVA and Tukey’s test, *n* = 2, COL vs. PBS *p*-value < 0.05), whereas in PEMF-treated animals, the YAP levels increased (ANOVA and Tukey’s test, *n* = 2, COL + PEMF vs. COL *p*-value < 0.01). The increment, although not significant, was seen also when PEMF treatment started on day 15 (Figure 3F). PEMF treatment also restored the levels of PGC1alpha (Figure 3G), which were decreased by inflammation (ANOVA and Tukey’s test, *n* = 2, COL vs. PBS *p*-value < 0.05; COL + PEMF vs. COL *p*-value < 0.001).

### 2.4. Muscle Fiber Characterization

**Figure 4:**
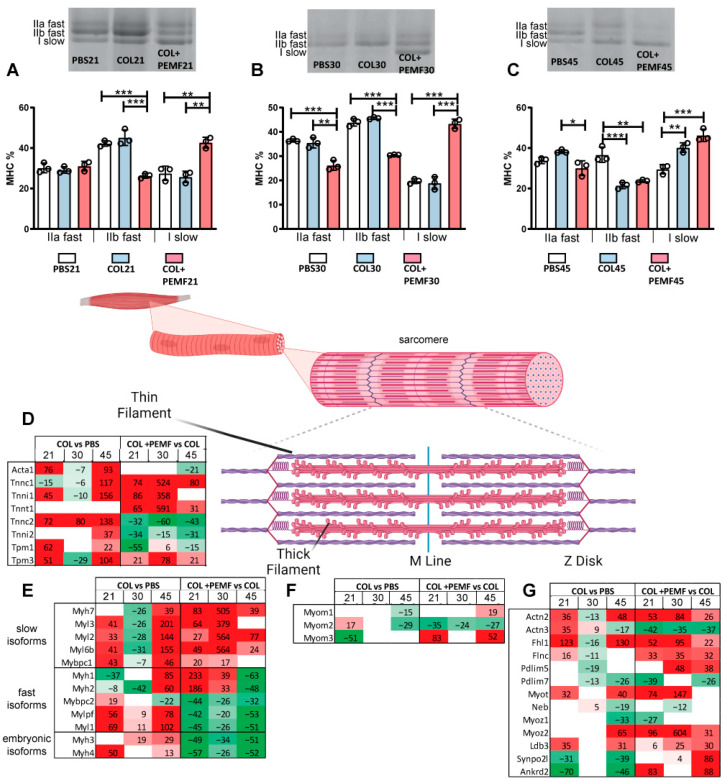
Muscle fiber characterization and contractile proteins. (**A**–**C**) Bar graphs showing the distribution of myosin heavy chain (MyHC) isoforms in untreated and PEMF-treated animals sacrificed at 21 days (**A**), 30 days (**B**), and 45 days (**C**) after collagenase injection. ANOVA and Tukey’s test, *n* = 3, * *p*-value < 0.05; ** *p*-value < 0.01; *** *p*-value < 0.001. Representative gel images are displayed. (**D**–**G**) Heatmaps illustrating the expression profile and % fold changes in significantly increased (in red) or decreased (in green) proteins (ANOVA and Tukey’s test, *p*-value < 0.05) in sarcomere structures: thin filaments (**D**), thick filaments (**E**), M line (**F**), and Z disk (**G**). Full-length images are available in Supplementary Figure S2. The graphical illustration was generated using BioRender (version 4).

Concerning muscle fiber distribution and changes in contractile proteins, myosin heavy chain (MyHC) isoform distribution was assessed in the three groups at all time points. After 15 days of PEMF exposure (Figure 4A), the percentage of type IIb fibers (fast) decreased, whereas type I fibers (slow) significantly increased compared to the PBS and COL groups. When PEMF exposure started 15 days after the lesion (Figure 4B), type IIa fibers (fast) also decreased, whereas prolonged exposure (15 + 30 days) (Figure 4C) reduced the gap between the COL and COL + PEMF groups.

### 2.5. Contractile Proteins

Concerning the muscle contractile machinery, differentially expressed proteins were organized following the muscle structure in the context of their role in muscle contraction. Proteins from the thin filaments characterizing slow-type fibers, such as troponin C1 (Tnnc1) and I1 (Tnni1), were increased in the muscle from injured limbs after 45 days, while they were increased earlier in PEMF-treated rats. Conversely, fast-type troponins, such as troponin C2 (Tnnc2) and I2 (Tnni2), were decreased after PEMF treatment in COL muscle (Figure 4D). MYH7, corresponding to myosin slow (MHC-slow), increased after 45 days in COL muscle, whereas the increment was observed at all time points in PEMF-treated rats, whereas MYL3, MYL2, MYL6B, MYLBPC1 light chains, and MyH4 (MyHC-2B) increased in both treated and untreated animals although to different extents. Thick filament fast isoforms increased after 21 and 45 days but were decreased after PEMF treatment, except for Myh1 (MyHC-IIX fast) and Myh2 (MyHC-II fast) that increased in COL after 45 days and increased in PEMF vs. COL after 15 and 30 days of treatment. The embryonic isoform of MYH3 increased in COL-treated animals after 30 and 45 days, whereas it decreased at all time points after PEMF treatment. (Figure 4E). Myomesin 2 (Myom 2) decreased after PEMF exposure, whereas Myom3 increased (Figure 4F). Z-band proteins increased in COL21 vs. PBS21 and in COL45 vs. PBS45 comparisons, except Pdlim7, Neb, Myoz1 Synpo2l, and Ankrd2. After PEMF treatment, Actn2, Fhl1, Flnc, Pdlim5, Myot, Myoz2 (slow), Sympo2l, and Ankrd2 increased, whereas Actn3, Pdlim 7, Neb, and Myoz1 (fast) decreased (Figure 4G).

### 2.6. Structural Proteins

Table 2 shows the results from the ECM and cytoskeletal compartments. Among extracellular matrix proteins, Col1a1 and Col1a2 strongly increased after 45 days in the muscle from injured limbs, as did Col6a2, Dcn, and Fbn1. In PEMF-treated groups, a general decrease in these proteins was observed, except for osteoglycin (Ogn).

Among cytoskeletal proteins, actin beta (Actb), desmin (Des), and PDZ and LIM Domain 3 (Pdlim3) increased in COL vs. PBS, whereas plectin (Plec), Des, and tubulin alpha 4a (Tuba4a) increased in COL + PEMF- vs. COL-treated rats.

### 2.7. Calcium Binding Proteins

Table 3 reports the calcium binding protein fold changes. Pvalb had an opposite trend in COL and in COL + PEMF muscles, as for Casq2 and Slc25a11 and SERCA. SERCA 1 (Atp2a1), the fast-twitch isoform, increased in COL but decreased in COL + PEMF. The low-abundant slow-twitch isoform of SERCA 2 (Atp2a2) increased in COL + PEMF21 compared to COL21 and decreased in COL + PEMF30 compared to COL30. SERCA 3 (Atp2a3) decreased in COL muscles and increased in COL + PEMF30 compared to COL30.

Transport proteins, proteins related to chromatin organization and proteostasis, and proteins regulating membrane curvature and remodeling are listed in Supplementary Table S6. In addition, Table S7 reports the fold changes in the comparison between PEMF-treated vs. untreated left counterparts (PBS + PEMF vs. PBS) to determine if the electromagnetic fields have the same effect on the inflamed limb and on the control limb.

### 2.8. Diseases and Biofunctions

Disease and biofunctions analysis conducted by IPA software highlighted the inhibition of muscle cell death, necrosis, and apoptosis in PEMF-treated groups (with a z-score lower than −2 in all groups), as well as the presence of reactive oxygen species (Table 4). The increase in lipids and fatty acid metabolism observed in the COL vs. PBS comparison (z-scores were 3.26 and 2.51, respectively, in COL21 vs. PBS21 comparison) was counteracted by PEMF treatment with a decrease in lipid and fatty acid levels and an increase in lipid oxidation. The mitochondrial transmembrane potential and synthesis of ATP were decreased in injured limbs, but the respiration of mitochondria increased after PEMF treatment. Interestingly, fibrosis and the production of lactic acid were decreased in PEMF-treated groups (z-score were −3.23 and −2.19, respectively, in COL + PEMF21 vs. COL21 comparison), whereas the level of Ca^2+^ increased (z-score = 2.19 in COL + PEMF21 vs. COL21 comparison and 2.36 in COL + PEMF45 vs. COL45 comparison).

## 3. Discussion

Regeneration in muscle occurs physiologically via multiple signaling pathways in an evolving regulation of the contractile machinery and energy homeostasis. Tendon disorders often involve not only tendon tissue but the entire tendon–bone–muscle unit. Therefore, adequate treatment would promote a physiological restoration of the entire unit. The above results describe *gastrocnemius* muscle changes upon experimental tendinopathy induced by type I collagenase in young rats and the recovery after exposure to 1.5 mT and 75 Hz PEMFs for 8 h per day. The treatment started on day 7 or 15 after the induction of the pathology and was administered for 15 or 30 days. The effects of PEMF treatment on the muscle proteome were compared with pathological but untreated muscle collected 21, 30, and 45 days after collagenase injection. Considering the entire set of changed proteins, muscles without any intervention showed protein changes that progressively increase over time, while treated animals showed increased changed proteins until day 15, and no further increase was observed after prolonging PEMF treatment.

Concerning protein changes, proteins regulating muscle metabolism were influenced both in the presence or absence of PEMF treatment, although in the opposite direction. A strong increment in glycolytic enzymes, particularly on day 21, retained on day 30 and 45, characterized untreated animals. In the same groups, the only decreased enzyme of the glycolytic pathway was LDHB, particularly on day 21. Changes were retained until day 30 and normalized on day 45, indicating that 45 days are required to recover the glycolytic pathway. Conversely, PEMF treatment caused the inhibition of the glycolytic pathway irrespective of the treatment duration, and the only enzyme that was strongly increased, particularly on day 30, was LDHB. LDHB and LDHA have a higher affinity for lactate; the former preferentially converts lactate to pyruvate and NAD+ to NADH when oxygen is abundant, and the latter converts pyruvate to lactate and NADH to NAD+ mainly in anaerobic conditions [32]. It can be postulated that in PEMF-treated animals the increase in LDHB becomes a source of NADH promoting mitochondrial respiration, whereas it acts as a gluconeogenic precursor in untreated animals [33]. Since LDHB increases respiratory chain activity, the overproduction of ROS was expected in PEMF-treated animals, but it was quenched by the action of antioxidant enzymes such as superoxide dismutase, catalase, and glutathione peroxidase, as described also by Maiullari S. et al. [34]. It is known that LDHB can act as a signaling molecule, modulating calcium levels and activating antioxidant enzymes [35,36]. NAD signaling impacts also at the nuclear level, as indicated by the TEAD1 upstream regulator identified by the IPA analysis and confirmed by the YAP increment in treated rats, suggesting a positive role of YAP in inducing a protective hypertrophy in homology with results described by Toshihide Kashihara in cardiac tissue [37]. By comparing the protein expression of treated and untreated rats, changes in the metabolic enzymes of the TCA cycle were observed. After 21 days, most of the TCA enzymes were decreased in untreated animals, indicating a metabolic impairment only partially recovered on days 30 and 45. Conversely, PEMF treatment increased TCA enzymes, and the increment was retained also after 30 days. In particular, the increase in Mdh and Sdhb by PEMF stimulation aligns with findings from a study on diabetic rats, where PEMFs restored the levels of SDH and MDH that were decreased by diabetes [38]. Additionally, in insulinoma cells, exposure to PEMF attenuated insulin secretion by affecting calcium influx through calcium channels [39], suggesting metabolic and physiological effects of PEMF stimulation. Lipid metabolism was strongly upregulated after PEMF treatment, such as enzymes regulating fatty acid metabolism. Of relevance was the activation of the muscle isoform of a transmembrane enzyme of the mitochondrial outer membrane CPT1B, which converts long-chain acyl-CoA to acylcarnitine, which enters the mitochondrial matrix promoting beta-oxidation and decreases the production of triglycerides, diacylglycerols, and ceramides [40]. Untreated muscle recovered lipid metabolism only partially, and CPT1B remained decreased also on day 45.

These effects were supported by an increased level of PGC1alpha in PEMF-treated rats, confirming our proteomic observation. PGC1alpha is a regulator for oxidative muscle remodeling and elevates mitochondrial biogenesis, promoting a shift from glucose to fatty acid as an energy source coordinating each step required for ATP synthesis [41,42]. Furthermore, the remodeling of muscle tissue to a fiber-type composition that is metabolically more oxidative and less glycolytic and its participation in the regulation of both carbohydrate and lipid metabolism [43] is supported by the pattern of fiber-type distribution in untreated and treated animals. In a recent paper, it was described that a brief exposure to low mT-Pemf in rats increases in vitro myogenesis and mitochondriogenesis by activating a calcium–mitochondrial axis upstream of PGC-1α transcriptional upregulation, recapitulating the response associated with exercise-induced metabolic adaptations [30]. Our results suggest that in rats under PEMF treatment, PGC1alpha overproduction was promoted by NAD signaling [44,45]. Also, YAP1-TEAD signaling is necessary for PGC1alpha expression and mitochondrial biogenesis modulation both in vitro and in vivo [34]. The Hippo signaling pathway plays an important role in mediating oxidative stress and induces cell death [46]. It has been described that a loss of Tead1 expression in adult cardiomyocytes increased mitochondrial ROS and disrupted the structure of mitochondria, leading to necroptosis [47].

Looking at contractile and structural proteins, the results indicated that collagenase increased proteins associated with thin filaments inducing an increase in actin, troponin, and tropomyosins particularly on day 45. In PEMF-treated animals, this set of proteins increased variably with unchanged or decreased levels of actin, increased level of isoforms of troponins and tropomyosins characterizing slow fibers (Tnnc1, Tnni, Tnnt, and Tpmn3), and unchanged or decreased levels of Tnnc2, TnnI2, and Tpm1, isoforms characterizing fast fibers. The decreased levels of fast-fiber-specific proteins, creatine kinase, and parvalbumin confirmed the changes observed in the fiber-type distribution [48]. The expression of myosins could be modulated by hypertrophic and metabolic signals. In the thick filaments, several slow myosin isoforms increased in untreated animals on day 45, as highlighted also by the myosin isoform distribution, indicating that the regeneration of thick filaments occurs only after 45 days, promoted by the overexpression of the embryonic isoform Myh3. These changes were not present in PEMF-treated rats in which an increase in slow myosin isoforms characterized all groups and was more significant in COL + PEMF30 compared to COL30. Slow myosin Myh7 strongly increases after PEMF treatment on day 30, confirming the switch towards oxidative metabolism highlighted by the isoform distribution. Fast isoforms of myosins of the thick filament were increased in COL45 vs. PBS45, whereas they were decreased in all PEMF-treated animals, except for Myh1 (type IIX) and Myh2 (type IIA) that, although being classified as fast isoforms, are associated with oxidative mechanisms [49,50]. The effects of PEMF exposure on enhancing oxidative muscle capacity were described also by Kit Tai et al. [30] in mice, together with increased Pgc-1α, fatty acid oxidation, and reduced insulin levels, with beneficial effects on the gut microbiome. In treated animals, Myom3 increased, indicating that the alignment of fibers [51,52] for the proper transmission of contraction is active. The Z-band ensures the transmission of tension from one sarcomere to the next; the proper transmission was indicated by the increment in several proteins located in the Z-band that induce a slight positive regulation of RhoGDI protein signal transduction, promoting fiber alignment in PEMF-treated animals, whereas it decreased at all time points in COL-treated animals [53]. In treated animals, a positive regulation of the stress fiber assembly and sarcomere organization (Synpo2L and Ankyrin, Myot) and of Myoz 2, contributing to calcineurin/alpha-actinin tethering at the z-line, was observed, whereas in untreated animals, a less efficient transmission seemed to occur. Unfortunately, functional tests were not included in this study originally designed to assess the recovery of the Achilles tendon [23] and the impact at the molecular level on the *gastrocnemius*.

Cytoskeletal proteins varied, with an increase in proteins mediating the interlink of cytoskeletal elements like plectin, desmin, and new tubule formation (TUB) in treated animals suggesting that the ability of force transfer to the extracellular matrix at the costamere structure level was preserved. The ECM protein composition indicates an increase in collagen synthesis in untreated rats retained also on day 45 when fibrillogenesis may occur, as indicated by increased levels of Dcn and Fbn1. Conversely, in PEMF-treated rats, collagen synthesis is active in group COL + PEMF21 vs. COL21 only, whereas all ECM proteins are decreased except for the ubiquitous ECM component OGN, involved in fiber assembly and extracellular matrix organization [54]. Low-intensity electromagnetic fields induce a trophic stimulus [55]. The effect of PEMFs without tendinopathy was analyzed in the comparison between PEMF-treated vs. untreated left counterparts (PBS + PEMF vs. PBS). Although similar to the results of the comparison of the inflamed limbs (COL + PEMF vs. COL), due to hypertrophic effects, there were differences in the fold changes in the stress response proteins on day 45 and in contractile, extracellular matrix, and cytoskeletal proteins on days 21 and 45, indicating that the effect of PEMFs could be modulated by the presence of tendinopathy and tendon–muscle cross-talk.

Unfortunately, the cross-talk between the tendon and the myotendinous junction and extracellular matrix could not be detected by this study, but we know from a recent work on the same rats and with the same experimental setting that PEMF exposure significantly enhanced the recovery of the tendon structure and restored physiological cell morphology, with a decrease in hypercellularity and vascularity [23]. In the tendon, it has been demonstrated that PEMFs enhance the anti-inflammatory efficacy of adenosine through the adenosine receptors (ARs), promoting tendon damage recovery through a reduction in pro-inflammatory cytokines [23]. It is known that adenosine receptor activation in young mice increases lactate efflux and improves cardiac function [56]. Adenosine A2BRs are highly expressed in skeletal muscle, and mice with muscle deletion of A2B exhibited sarcopenia, diminished muscle strength, and reduced energy expenditure and could be targeted to counteract sarcopenia [57]. Adenosine and adenosine receptors were also associated with glucose clearance and lipolysis, whereas glycogen synthesis is not stimulated by adenosine, suggesting that in physiological recovery adenosine and adenosine receptor stimulation were not involved (supported by increased level of adenosine deaminase 1) [58,59]. Conversely, based on our results, we can postulate that in young rats LDHB and NADH production can promote ATP synthesis and adenosine delivery with adenosine receptor activation in PEMF-treated rats. The activation of this signaling contributes to muscle recovery in young PEMF-treated rats, while the physiological recovery in untreated animals is delayed and is not reached even after 45 days.

In conclusion, our data show that PEMF treatment can positively contribute to muscle recovery after tendinopathy by acting on metabolic pathways, such as NAD signaling and oxidative phosphorylation, increasing PGC1alpha and YAP. After PEMF exposure, myosins regulating slow-twitch fibers increased, as well as cytoskeletal and z-disk proteins involved in fiber alignment and force transmission, accelerating physiological recovery. This study is the first to investigate muscle proteomic changes induced by tendinopathy and PEMF treatment, shedding light on muscle atrophy and wasting following tendon disorders and showing translational potential for restoring muscle functionality. However, further studies are needed to translate the benefits of PEMF treatment for muscle into clinical practice, given that this research was limited to an animal model subjected to whole-body PEMF stimulation for 8 h per day. Additionally, a post-treatment muscle proteomic analysis would be beneficial to determine if the changes in the muscle proteome observed after PEMF treatment are retained over time, as suggested by our data indicating functional recovery.

## 4. Materials and Methods

### 4.1. Animal Housing and Ethic Statements

This study is reported in accordance with the ARRIVE guidelines, and it was conducted in adherence with the principles and laws, regulations, and policies governing the care and use of laboratory animals, the NIH Guide for the Care and Use of Laboratory Animals (2011 edition), and EU directives and guidelines (EEC Council Directive 2010/63/UE).

Animals were housed in a dedicated facility with light and dark cycles every 12 h. Animal health, animal welfare, the experimental protocols, and the procedure were monitored by a certified veterinary doctor over the study duration. All surgeries were performed under general anesthesia, as previously described [24,60].

### 4.2. Study Design

Sprague Dawley rats were unilaterally induced for tendinopathy by injecting 3 mg/mL of type I collagenase (Clostridium histolyticum 185 IU/mg; Worthington, Lakewood, NJ, USA) dissolved in sterile saline solution (COL group) in right limbs, as previously described [19], while the left sides were injected with PBS (PBS group). Muscles from rats that did not receive PEMF treatment were named COL21, COL30, and COL45 and PBS21, PBS30, and PBS45 according to the sacrifice day (after 21, 30, and 45 days, respectively). Treated animals were exposed to PEMF for 15 days starting on day 7 or on day 15 or for 30 days starting on day 15, and the muscles from the limbs induced for tendinopathy and treated with PEMFs were named COL + PEMF21, COL + PEMF30, and COL + PEMF45, respectively, whereas the muscles from control limbs were named PBS + PEMF21, PBS + PEMF30, and PBS + PEMF45 (Table 1).

PEMF treatment (1.5 mT SD 0.2; 75 Hz) was administered for eight hours/day. A custom-made coil (40 cm × 18 cm) was placed at the bottom of the rat cages and connected to a PEMF generator system (IGEA SpA, Carpi, Italy) which imposed a uniform electromagnetic field across the volume of the cage [24]. Then, the animals were sacrificed at the selected time points, and the *gastrocnemius* muscles were removed from the limbs and stored at −80 °C until processing.

### 4.3. Protein Extraction

*Gastrocnemius* muscles were crushed in a frozen mortar, suspended in lysis buffer [7 M urea, 2 M thiourea, 4% CHAPS, 30 mM Tris, 1 mM PMSF, 1% phosphatase inhibitor cocktail 1 and 2 (Sigma), pH 8.5], and solubilized by sonication on ice. Proteins were selectively precipitated using the PlusOne 2D-Clean up kit (GE Healthcare, Chicago, IL, USA) in order to remove non-protein impurities and resuspended in 50 mM ammonium bicarbonate and 0.1% RapiGest SF surfactant (Waters). Dithiotreitol (DTT) was added to a final concentration of 5 mM for cysteine reduction, and samples were incubated for 30 min at 60 °C. Iodoacetamide (IAA) was added to a final concentration of 15 mM and incubated for 30 min in the dark. Proteins were digested with trypsin (Promega) using an enzyme/protein ratio of 1:50 at 37 °C overnight. On the next day, the digestion was stopped and RapiGest precipitated by adding trifluoroacetic acid (TFA) to a final concentration of 0.5%. After centrifugation at 13,000 rpm for 10 min, the supernatants were recovered, and the peptide concentration was determined by the Pierce™ Quantitative Colorimetric Peptide Assay (Thermo Scientific, Waltham, MA, USA).

### 4.4. LC-MS/MS Analysis

LC-ESI-MS/MS analysis was performed on a Dionex UltiMate 3000 HPLC System with an Easy Spray PepMap RSLC C18 column (250 mm, internal diameter of 75 μm) (Thermo Scientific). Gradient: 5% ACN in 0.1% formic acid for 10 min, 5–35% ACN in 0.1% formic acid for 139 min, 35–60% ACN in 0.1% formic for 40 min, 60–100% ACN for 1 min, and 100% ACN for 10 min at a flow rate of 0.3 μL/min. The eluate was electrosprayed into an Orbitrap Tribrid Fusion (Thermo Fisher Scientific, Bremen, Germany) through a nanoelectrospray ion source (Thermo Fisher Scientific). The LTQ-Orbitrap was operated in positive mode in data-dependent acquisition mode to automatically alternate between a full scan (350–2000 *m*/*z*) in the Orbitrap (at resolution 60,000, AGC target 1,000,000) and subsequent CID MS/MS in the linear ion trap of the 20 most intense peaks from the full scan (normalized collision energy of 35%, 10 ms activation). Isolation window: 3 Da; unassigned charge states: rejected; charge state 1: rejected; charge states 2+, 3+, and 4+: not rejected; dynamic exclusion enabled (60 s, exclusion list size: 200). Three technical replicates for each sample were acquired. Mass spectra were analyzed using MaxQuant software (version 1.6.3.3). The initial maximum allowed mass deviation was set to 6 ppm for monoisotopic precursor ions and 0.5 Da for MS/MS peaks. Enzyme specificity was set to trypsin/P, and a maximum of two missed cleavages were allowed. Carbamidomethylation was set as a fixed modification, while N-terminal acetylation and methionine oxidation were set as variable modifications. The spectra were searched by the Andromeda search engine against the Rattus Norvegicus Uniprot sequence database (release 22 October 2018). Protein identification required at least one unique or razor peptide per protein group. Quantification in MaxQuant was performed using the built-in XIC-based label-free quantification (LFQ) algorithm using fast LFQ. The required false positive rate (FDR) was set to 1% at the peptide, 1% at the protein, and 1% at the site-modification level, and the minimum required peptide length was set to 7 amino acids.

### 4.5. Bioinformatic Analysis

Bioinformatic analysis was carried out by Ingenuity Pathway Analysis (IPA) (QIAGEN Bioinformatics. Ingenuity Pathway Analysis). The quantitative protein data were imported into IPA software (version 22.0.2) to identify the protein–protein interactions, canonical pathways, upstream regulators, diseases and biofunctions, and networks most strongly associated with the protein list. The software uses experimental expression data on networks constructed from published interactions to give a score. A score greater than three (*p* ≤ 0.001) indicates a greater than 99.9% confidence that a protein network was not generated by chance. The results were filtered by a significant z-score (>2 if the pathway is activated or <2 if the pathway is inhibited).

### 4.6. Immunoblotting

Protein extracts (50 μg) from pooled PBS, COL, and COL + PEMF muscles were loaded in duplicate and resolved on 10–16% or on 5–10% gradient polyacrylamide gels, according to protein molecular weight. Blots were incubated with rabbit anti-PGC-1alpha or mouse anti-YAP polyclonal primary antibodies (Santa Cruz Biotechnology, Dallas, TX, USA, dilution 1:500). After washing, membranes were incubated with anti-rabbit (GE Healthcare) or anti-mouse (Santa Cruz Biotechnology) secondary antibodies conjugated with horseradish peroxidase. Signals were visualized by chemiluminescence using the ECL Prime detection kit and the Image Quant LAS 4000 (GE Healthcare) analysis system. Band quantification was performed using the Image Quant TL 10.2 (Molecular Dynamics) software followed by statistical analysis (ANOVA + Tukey, *p* < 0.05) (for full-length blot images, see Supplementary Figure S1). Band intensities were normalized against the total amount of proteins stained with Sypro ruby total-protein blot stain (Thermo Fisher Scientific).

### 4.7. MyHC Fiber Composition

SDS electrophoresis, from pooled muscle extracts, was performed in a discontinuous buffer system with a 4%T stacking gel, pH 6.8 and a 6%T, constant concentration, 37% *w*/*v* glycerol, pH 8.8, and running gel. Samples (3 ug) were separated at 100 V, overnight. Gels were stained with SYPRO Orange (molecular probes) and scanned at 570 nm with a Ettan DIGE Imager (GE Healthcare). Quantitation was achieved using ImageQuant v10.1 (Molecular Dynamics) software. Full-length images are available in Supplementary Figure S2.

### 4.8. Data Analysis

The statistical analyses on LFQ data were performed using the Perseus software (version 1.6.1.3). Only proteins present and quantified in at least 2 out of 3 technical repeats were considered as positively identified in a sample and used for statistical analyses. ANOVA tests (permutation-based FDR < 0.05) were carried out to identify proteins differentially expressed among the different conditions.

## Figures and Tables

**Table 1:** Animal grouping and details of PEMF exposure start time and duration.

Group Name	Muscle Group Name	Number of Samples	Type I Collagenase Injection	PEMF Treatment	Start of PEMF Treatment (Days after Collagenase Injection)	Length of Treatment (Days)	Sacrifice and Muscle Extraction
	PBS21	3	NO	NO	--	--	Day 21
PBS	PBS30	3	NO	NO	--	--	Day 30
	PBS45	3	NO	NO	--	--	Day 45
	COL21	3	YES	NO	--	--	Day 21
COL	COL30	3	YES	NO	--	--	Day 30
	COL45	3	YES	NO	--	--	Day 45
	PBS + PEMF21	3	NO	YES	Day 7	15	Day 21
PBS + PEMF	PBS + PEMF30	3	NO	YES	Day 15	15	Day 30
	PBS + PEMF45	3	NO	YES	Day 15	30	Day 45
	COL + PEMF21	3	YES	YES	Day 7	15	Day 21
COL + PEMF	COL + PEMF30	3	YES	YES	Day 15	15	Day 30
	COL + PEMF45	3	YES	YES	Day 15	30	Day 45

**Table 2:** Expression profile and % fold changes in significantly changed proteins (ANOVA and Tukey’s test, *p*-value < 0.05) of extracellular matrix and cytoskeleton.

			COL vs. PBS			COL + PEMF vs. COL		
			21 d	30 d	45 d	21 d	30 d	45 d
Extracellular matrix	P02454	Col1a1	133.38	−28.93	611.14	16.77	−65.17	−85.31
	F1LS40	Col1a2	169.30	15.46	809.56	47.85	−81.78	−91.15
	D3ZUL3	Col6a1	28.86	−21.04			−21.69	−24.96
	F1LNH3	Col6a2		−27.56	88.99			−29.89
	D3ZZT9	Col14a1					−31.25	
	Q9EQP5	Prelp		−49.97				
	G3V6E7	Fmod		−68.85				−48.48
	P51886	Lum					−29.79	
	P11762	Lgals1					−14.03	
	D3ZVB7	Ogn	−10.65	−46.80	−62.60		75.52	44.45
	Q5XIH1	Aspn		29.74			−35.05	
	Q01129	Dcn		−38.72	77.06			−27.85
	G3V9M6	Fbn1			139.39	−34.12		
Cytoskeletal	P60711	Actb		−21.87	151.82			−29.33
	G3V8C3	Vim	−66.07		−40.97			−26.47
	F7F9U6	Plec	−25.41	35.01	−33.90		43.35	40.22
	Q6P725	Des	30.10		51.11	34.94	26.35	
	Q6P9V9	Tuba1b		−8.67			−13.82	
	Q5XIF6	Tuba4a	−20.32	−18.15	−25.49	15.73	27.77	56.35
	G3V7C6	Tubb4b	−21.14	−19.46			27.07	
	A0A0G2JSM3	Pdlim3	72.58		71.12		−28.54	
	G3V6P7	Myh9			−39.31			9.20
	G3V9G5	Synm				−22.32		32.49
	P11530	Dmd			−50.43			−35.62

**Table 3:** Expression profile and % fold changes in significantly changed calcium binding proteins (ANOVA and Tukey’s test, *p*-value < 0.05).

			COL vs. PBS			COL + PEMF vs. COL		
			21 d	30 d	45 d	21 d	30 d	45 d
Calcium binding	P02625	Pvalb	−57.84	260.72	23.61	24.72	−82.80	−90.03
	F1LWG8	Srl		−17.33		−17.16	12.29	
	P19633	Casq1		−10.17	−38.84	−48.52		
	F1M944	Casq2	−53.03			60.38		124.76
	F1LMY4	Ryr1	−31.99		−69.65	−49.24		
	Q6P9Y4	Slc25a4	20.20			15.28		29.32
	G3V6H5	Slc25a11	−43.44		−51.29	39.97		39.16
	F1LX07	Slc25a12	−39.19	−16.48	−50.96			
	Q64578	Atp2a1	88.39	17.38	73.89	−28.80	−55.07	−56.95
	P11507	Atp2a2			14.99	77.83	−36.49	
	A0A0G2K9N9	Atp2a3	−58.84	−43.85			45.25	

**Table 4:** Heatmap displaying the most significant disease and biofunctions results. The orange- and blue-colored rectangles indicate predicted activation or inhibition, respectively, via the z-score statistic.

Diseases and Biofunctions	COL vs. PBS			COL + PEMF vs. COL		
	21 d	30 d	45 d	21 d	30 d	45 d
Cell death of muscle cells	1.70	1.03	0.24	−**2.50**	−**2.43**	−**3.08**
Necrosis of muscle	1.02	1.30	0.02	−**2.65**	−**2.23**	−**2.71**
Apoptosis of muscle cells	1.53	1.19	−0.18	−**2.58**	−**2.16**	−**2.75**
Necrosis	1.49	0.95	0.67	−**2.75**	−0.81	−**2.03**
Apoptosis	**2.20**	1.10	−0.05	−**3.22**	−1.35	−1.56
Survival of stem cell lines	−1.13	N/A	N/A	N/A	**2.83**	**2.24**
Quantity of reactive oxygen species	**2.62**	1.53	1.10	−**2.95**	−**2.82**	N/A
Synthesis of reactive oxygen species	1.29	1.20	0.95	−**2.62**	−**2.48**	−**2.03**
Metabolism of reactive oxygen species	0.61	0.93	0.46	−**2.08**	−1.87	−1.65
Concentration of lipid	**3.26**	0.57	0.67	−**2.35**	−1.86	−1.63
Concentration of fatty acid	**2.51**	0.89	0.96	N/A	−**3.05**	−**2.47**
Oxidation of lipid	−1.43	N/A	N/A	**2.15**	0.92	1.52
Fatty acid metabolism	1.09	−0.16	**2.12**	−0.04	0.40	−0.94
Transmembrane potential of mitochondria	−**2.60**	N/A	−**2.43**	1.39	1.53	1.25
Respiration of mitochondria	−1.41	N/A	−1.02	N/A	**2.00**	1.95
Synthesis of ATP	−0.85	−1.63	−**2.54**	0.77	0.85	−0.29
Fibrosis	−0.66	−0.35	−1.63	−**3.23**	0.18	−1.49
Production of lactic acid	**2.19**	N/A	N/A	−**2.19**	N/A	N/A
Quantity of Ca^2+^	N/A	N/A	N/A	**2.19**	N/A	**2.36**

## Data Availability

The datasets generated and analyzed during the current study are available in the OSF repository (https://osf.io/9qhxd/, accessed on 25 July 2024).

## Supplementary Materials

The following supporting information can be downloaded at https://www.mdpi.com/article/10.3390/ijms25168852/s1.
