# Supplementary material for: Effects of Pulsed Electromagnetic Field Treatment on Skeletal Muscle Tissue Recovery in a Rat Model of Collagenase-Induced Tendinopathy: Results from a Proteome Analysis
Source: Int J Mol Sci. 2024 Aug 14;25(16):8852. doi: 10.3390/ijms25168852 (PMC11354614; doi:10.3390/ijms25168852)
Supplement: Supplementary file 1 [file ijms-25-08852-s001.zip › Figure S1 S2.pptx]

## Slide 1
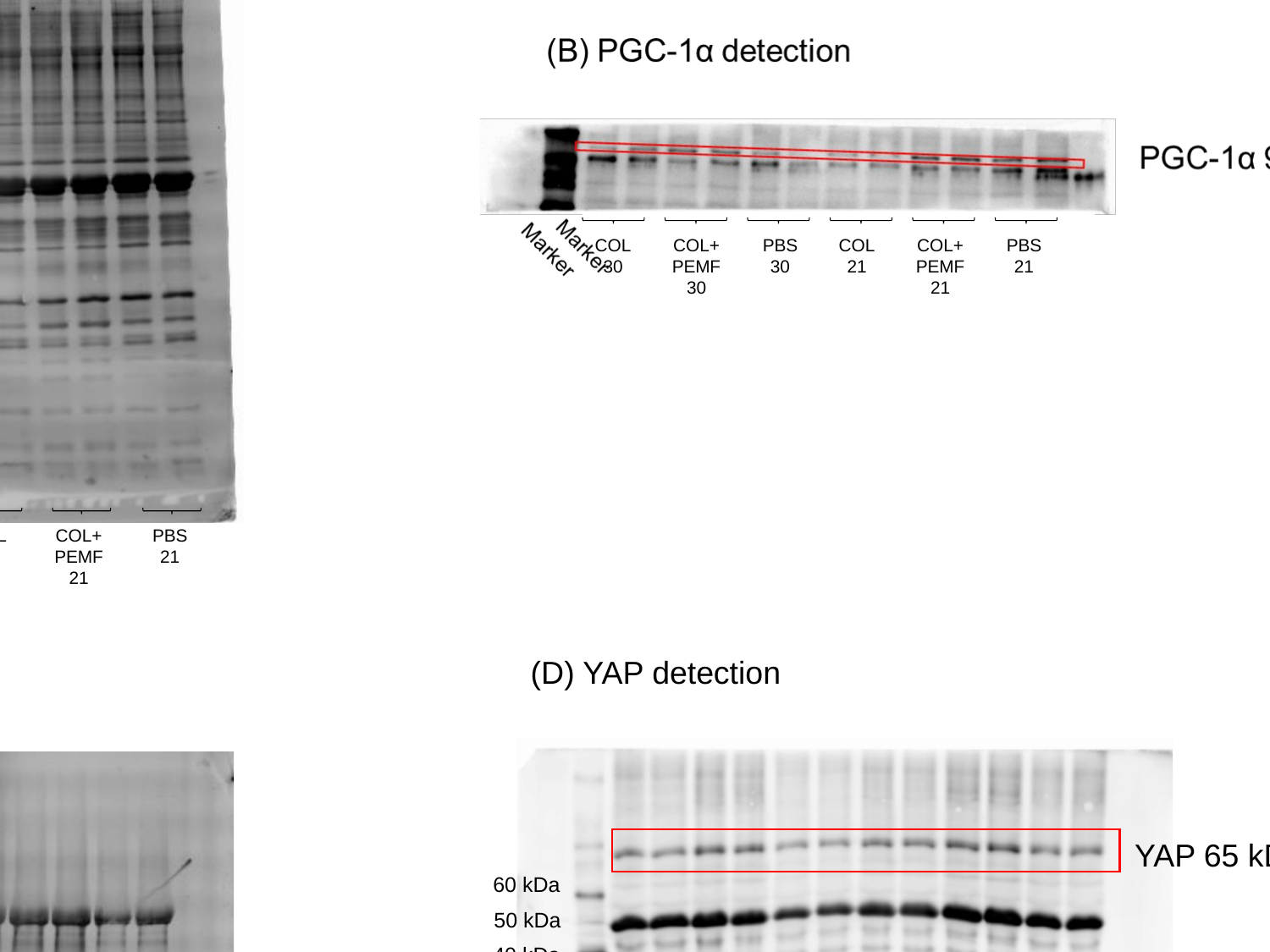

Figure S1
(A)
(C)
Marker
Marker
(D) YAP detection
YAP 65 kDa
60 kDa
50 kDa
40 kDa
30 kDa
Marker
Marker
COL
30
COL+
PEMF
30
PBS
30
COL
21
COL+
PEMF
21
PBS
21
COL
30
COL+
PEMF
30
PBS
30
COL
21
COL+
PEMF
21
PBS
21
Bc Bc E E Bp Bp Ac Ac D D Ap Ap
Marker
Marker
COL
30
COL+
PEMF
30
PBS
30
COL
21
COL+
PEMF
21
PBS
21
COL
30
COL+
PEMF
30
PBS
30
COL
21
COL+
PEMF
21
PBS
21

## Slide 2
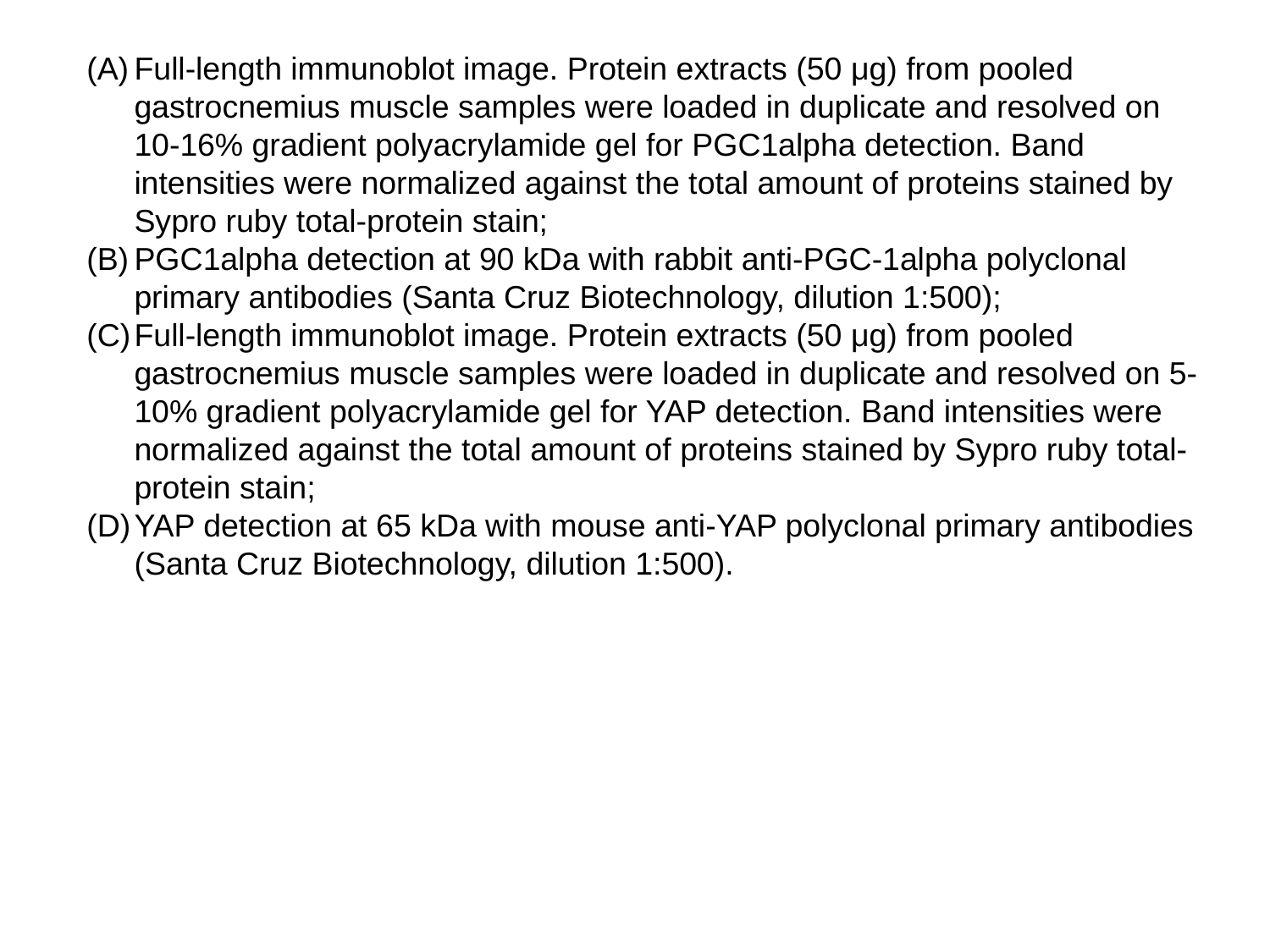

Full-length immunoblot image. Protein extracts (50 μg) from pooled gastrocnemius muscle samples were loaded in duplicate and resolved on 10-16% gradient polyacrylamide gel for PGC1alpha detection. Band intensities were normalized against the total amount of proteins stained by Sypro ruby total-protein stain;
PGC1alpha detection at 90 kDa with rabbit anti-PGC-1alpha polyclonal primary antibodies (Santa Cruz Biotechnology, dilution 1:500);
Full-length immunoblot image. Protein extracts (50 μg) from pooled gastrocnemius muscle samples were loaded in duplicate and resolved on 5-10% gradient polyacrylamide gel for YAP detection. Band intensities were normalized against the total amount of proteins stained by Sypro ruby total-protein stain;
YAP detection at 65 kDa with mouse anti-YAP polyclonal primary antibodies (Santa Cruz Biotechnology, dilution 1:500).

## Slide 3
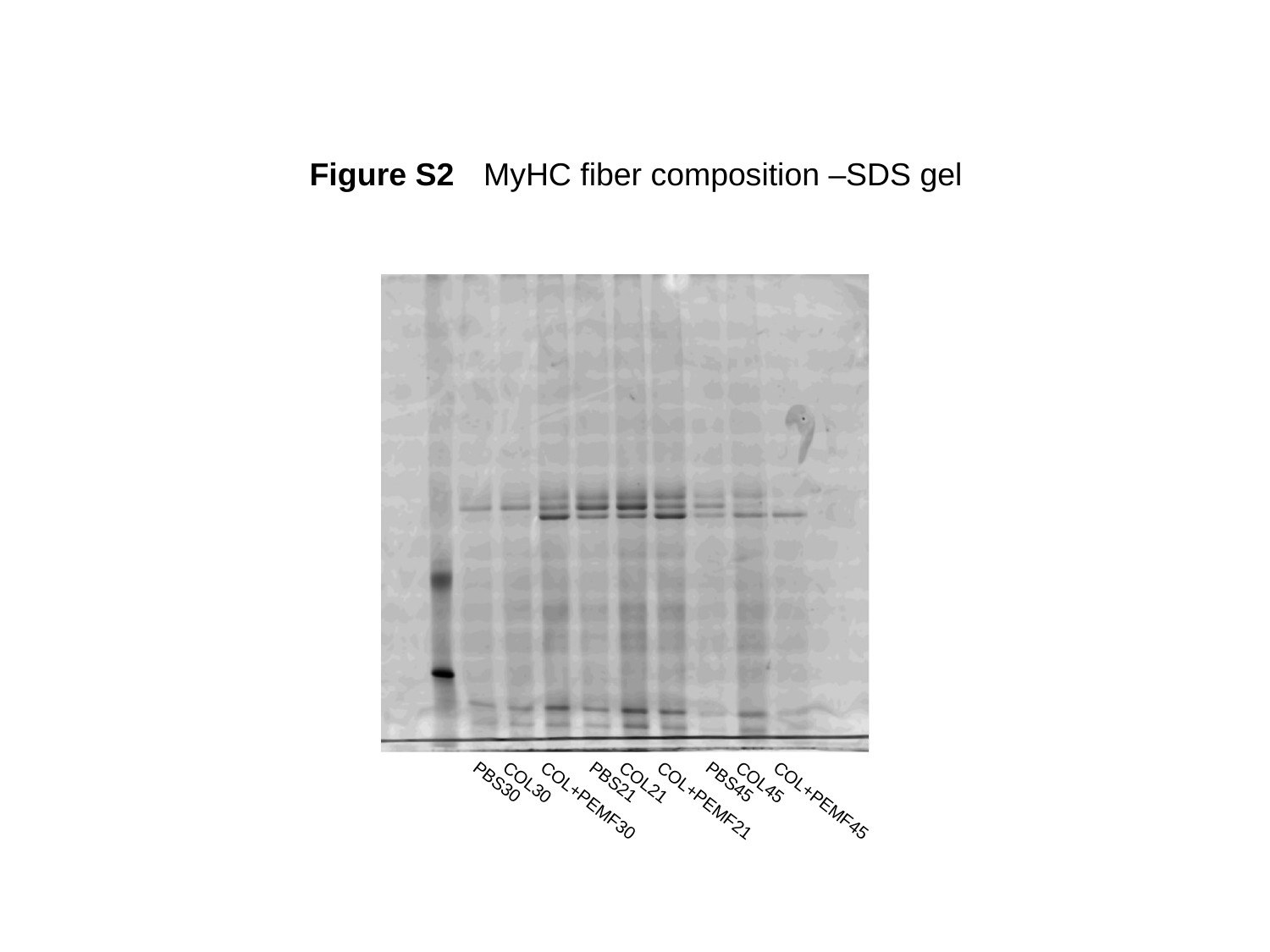

Figure S2
MyHC fiber composition –SDS gel
PBS30
PBS21
PBS45
COL30
COL21
COL45
COL+PEMF30
COL+PEMF21
COL+PEMF45

## Slide 4
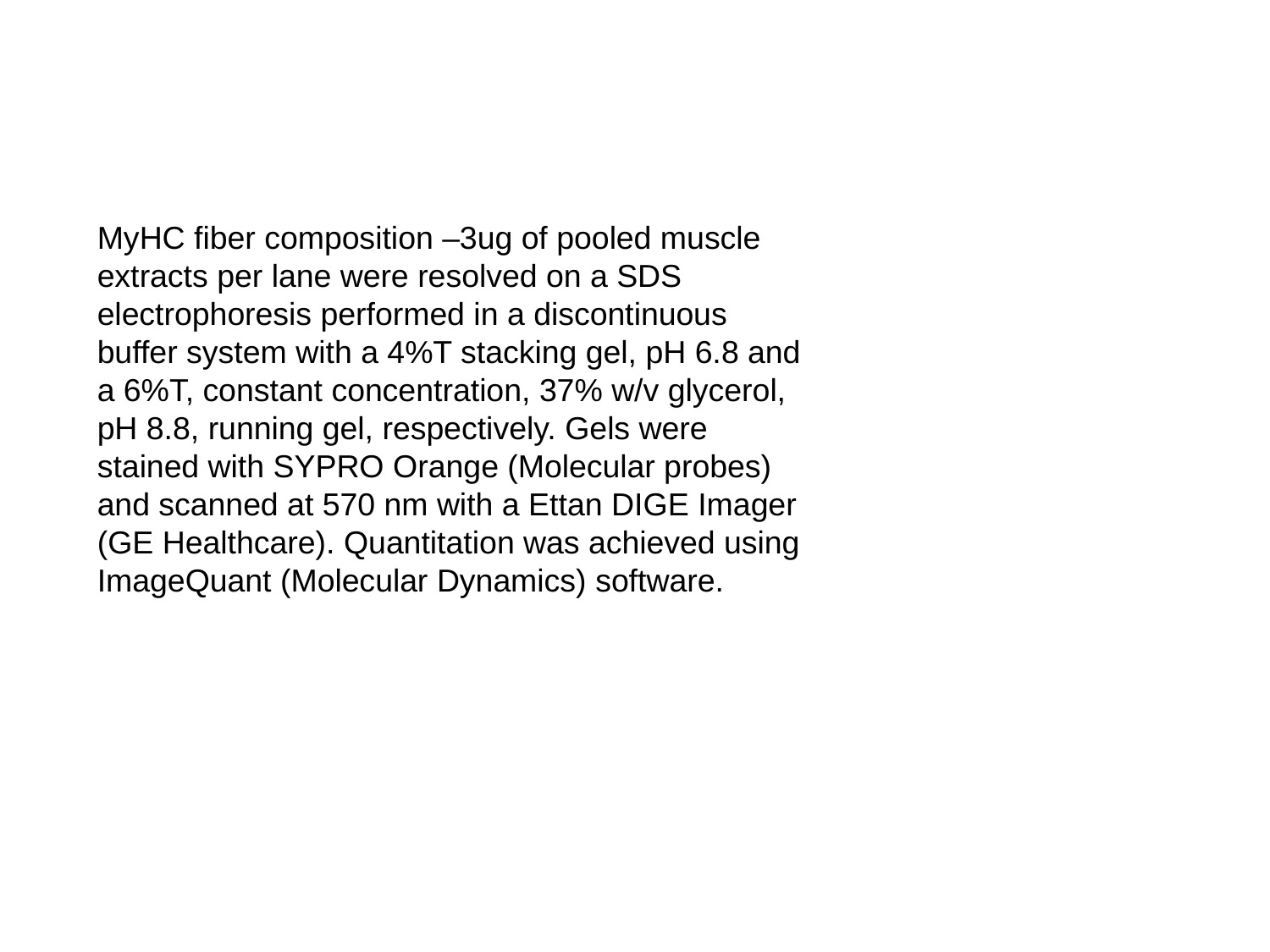

MyHC fiber composition –3ug of pooled muscle extracts per lane were resolved on a SDS electrophoresis performed in a discontinuous buffer system with a 4%T stacking gel, pH 6.8 and a 6%T, constant concentration, 37% w/v glycerol, pH 8.8, running gel, respectively. Gels were stained with SYPRO Orange (Molecular probes) and scanned at 570 nm with a Ettan DIGE Imager (GE Healthcare). Quantitation was achieved using ImageQuant (Molecular Dynamics) software.
